# Retraction note: Effect of platelet-rich plasma on the healing of cutaneous defects exposed to acute to chronic wounds: a clinico-histopathologic study in rabbits

**DOI:** 10.1186/s13000-016-0556-5

**Published:** 2016-11-02

**Authors:** Omid Ostvar, Sahar Shadvar, Emad Yahaghi, Kamran Azma, Amir Farshid Fayyaz, Koorosh Ahmadi, Iradj Nowrouzian

**Affiliations:** 1 Department of Pathology, Faculty of Veterinary Medicine, University of Tehran, Tehran, Iran; 2 Brain and Spinal Injury Research Center, Tehran University of Medical Sciences, Tehran, Iran; 3 Baqiyatallah University of Medical Sciences, Tehran, Iran; 4 Department of Physical Medicine and Rehabilitation, Clinical Biomechanical and Rehabilitation Engineering Research Center, AJA University of Medical Sciences, Tehran, Iran; 5 Department of Legal Medicine, AJA University of Medical Sciences, Tehran, Iran; 6 Department of Emergency Medicine, Alborz University of Medical Science, Karaj, Iran; 7 Department of Clinical Sciences, Faculty of Veterinary Medicine, University of Tehran, P.O. Box 14155–6453, Tehran, Iran

## Retraction

The Editor-in-Chief and Publisher have retracted this article [1] because the scientific integrity of the content cannot be guaranteed. An investigation by the Publisher found it to be one of a group of articles we have identified as showing evidence suggestive of attempts to subvert the peer review and publication system to inappropriately obtain or allocate authorship. This article showed evidence of plagiarism (most notably from the articles cited [2–5]) and authorship manipulation.

## References

[1] Ostvar O, Shadvar S, Yahaghi E, Azma K, Fayyaz AF, Ahmadi K, Nowrouzian I (2015). Effect of platelet-rich plasma on the healing of cutaneous defects exposed to acute to chronic wounds: a clinico-histopathologic study in rabbits. Diagn Pathol.

[2] Yang HS, Shin J, Bhang SH, Shin JY, Park J, Im GI, Kim CS, Kim BS (2011). Enhanced skin wound healing by a sustained release of growth factors contained in platelet-rich plasma. Exp Mol Med.

[3] Kimura A, Ogata H, Yazawa M, Watanabe N, Mori T, Nakajima T (2005). The effects of platelet-rich plasma on cutaneous incisional wound healing in rats. J Dermatol Sci.

[4] Carter CA, Jolly DG, Worden CE, Hendren DG, Kane CJ (2003). Platelet-rich plasma gel promotes differentiation and regeneration during equine wound healing. Exp Mol Pathol.

[5] Woo SH, Jeong HS, Kim JP, Koh EH, Lee SU, Jin SM, Kim DH, Sohn JH, Lee SH (2014). Favorable vocal fold wound healing induced by platelet-rich plasma injection. Clin Exp Otorhinolaryngol.

